# The synergy of β amyloid 1-42 and oxidative stress in the development of Alzheimer’s disease-like neurodegeneration of hippocampal cells

**DOI:** 10.1038/s41598-022-22761-5

**Published:** 2022-10-25

**Authors:** Gohar Karapetyan, Katarine Fereshetyan, Hayk Harutyunyan, Konstantin Yenkoyan

**Affiliations:** 1 Neuroscience Laboratory, Cobrain Center, Yerevan State Medical University named after M. Heratsi, 2 Koryun Str., 0025 Yerevan, Armenia; 2 Department of Biochemistry, Yerevan State Medical University named after M. Heratsi, Yerevan, Armenia

**Keywords:** Biochemistry, Neuroscience

## Abstract

Alzheimer’s disease (AD) is a type of dementia that affects memory, thinking and behavior. Symptoms eventually become severe enough to interfere with daily tasks. Understanding the etiology and pathogenesis of AD is necessary for the development of strategies for AD prevention and/or treatment, and modeling of this pathology is an important step in achieving this goal. β-amyloid peptide (Aβ) injection is a widely used approach for modeling AD. Nevertheless, it has been reported that the model constructed by injection of Aβ in combination with a prooxidant cocktail (ferrous sulfate, Aβ, and buthionine sulfoximine (BSO) (FAB)) best reflects the natural development of this disease. The relationship between oxidative stress and Aβ deposition and their respective roles in Aβ-induced pathology in different animal models of AD have been thoroughly investigated. In the current paper, we compared the effects of Aβ 1-42 alone with that of Aβ-associated oxidative stress induced by the FAB cocktail on the neurodegeneration of hippocampal cells in vitro. We constructed a FAB-induced AD model using rat primary hippocampal cells and analyzed the contribution of each compound. The study mainly focused on the prooxidant aspects of AD pathogenesis. Moreover, cellular bioenergetics was assessed and routine metabolic tests were performed to determine the usefulness of this model. The data clearly show that aggregated Aβ1-42 alone is significantly less toxic to hippocampal cells. Aggregated Aβ damages neurons, and glial cells proliferate to remove Aβ from the hippocampus. External prooxidant agents (Fe^2+^) or inhibition of internal antioxidant defense by BSO has more toxic effects on hippocampal cells than aggregated Aβ alone. Moreover, hippocampal cells fight against Aβ-induced damage more effectively than against oxidative damage. However, the combination of Aβ with external oxidative damage and inhibition of internal antioxidant defense is even more toxic, impairs cellular defense systems, and may mimic the late phase of AD-associated cell damage. Our findings strongly indicate a critical role for the combination of Aβ and oxidative stress in the development of neurodegeneration in vitro.

## Introduction

Alzheimer’s disease (AD) is a neurodegenerative disease that occurs in elderly individuals. Most patients show early loss of memory and progressively worsening of cognitive function, including language and mental impairments and loss of emotion and directionality^1,2^. With the development of society and changes in the human environment, the incidence of AD has increased year by year^3^. There is still no effective drug for treating AD.

Animal models are critical tools for better understanding the pathogenesis of AD and for investigating new therapeutic strategies. To date, the majority of transgenic AD mouse models exhibit the accumulation of β-amyloid (Aβ) plaques and neurofibrillary tangles^4,5^. However, alternative nontransgenic models are urgently needed, as indicated by the high failure rate of clinical trials on AD treatments^4^. This may be due to the complexity and multifactorial nature of AD pathogenesis. Oxidative stress and mitochondrial dysfunction are the main pathological features of AD^6,7^. Oxidative stress that occurs within the bilayer, hypothesized in the Aβ-induced oxidative stress hypothesis in which Aβ inserts as oligomers into the bilayer and serves as a source of reactive oxygen species (ROS)^8^. Oxidative stress in AD brain is manifested by decreased levels of antioxidant enzymes and also by increased protein oxidation (including protein carbonyls formation), lipid peroxidation, DNA oxidation, advanced glycation end products^9^. Mitochondria are the main consumers of oxygen and the main organelles responsible for the formation of ROS. ROS generation involves the spontaneous leakage of electrons from respiratory chain complexes and direct interaction of these electrons with oxygen molecules^10^. ROS attack proteins, lipids and nucleic acids, increasing the levels of end-products, such as protein carbonyls, thiobarbituric acid-reactive substances (TBARS), and malondialdehyde^11^. Additionally, mitochondria are unique organelles that provide ATP to the host cell by oxidative phosphorylation. Neurons are highly differentiated cells that require a high level of ATP to maintain an ionic gradient and generate potentials, as well as for neurotransmission^12^. Unlike other organs, the brain does not store excess ATP,thus, normal mitochondrial function is extremely important for the continuous generation and delivery of energy^13^.

In addition, neuronal cells are more vulnerable to ROS due to the high rate of oxygen consumption and the enrichment of polyunsaturated fatty acids in membranes^14,15^. Moreover, the level of redox metal ions is higher and the activity of the antioxidant system is lower in the brain than in other tissues^16^. There is a growing body of evidence showing the presence of markers of oxidative stress in neurofibrillary tangles and senile plaques (SPs) in AD brain. Several studies indicated significant elevation of membrane PUFA peroxidation by-product 4-hydroxynonenal (HNE) in hippocampus, amygdala, temporal cortex and cerebrospinal fluid of AD cases (W.R^17^. HNE neurotoxic mechanisms may be mediated by modifying neuron membrane proteins/enzymes, altered glucose and glutamate transport system, calcium metabolism, etc.^18^. This indicates that, in line with the primary damaging effect of reactive oxygen species on neuron membrane, a secondary, reaction by-products are capable of increasing neuron degeneration. Additionally, the level of antioxidant enzyme glutathione S-transferases (GST) is significantly decreased in AD brain^19^, consistent with the notion that a loss of protection against HNE might be correlated with subsequent protein modifications that lead to neuronal death. In fact, it is known that Aβ decreases activity of mitochondrial superoxide dismutase, which detoxifies the anion superoxide and protects from peroxidative damage^20^. Aβ is also capable of binding and inhibiting mitochondrial alcohol dehydrogenase known as ABAD (Aβ binding alcoholdehydrogenase)^21^. ABAD has a protective role, being responsible for the detoxification from aldehydes such as 4-hydroxynonenal.In addition, several studies have reported that Aβ affects mitochondrial DNA and proteins, leading to impairment of the electron transport chain and ultimately mitochondrial dysfunction. In turn, it should be noted that mitochondrial dysfunction causes alterations in amyloid precursor protein (APP) metabolism, enhances intraneuronal accumulation of Aβ and makes neuronal cells vulnerable^22^. Undoubtedly, the generation of ROS and accumulation of Aβ peptides are interconnected. It is important to note that alterations in the AD brain, such as the presence of redox metal ions, mitochondrial dysfunction, and an imbalance of antioxidant/oxidant enzymes, also induce ROS production^23^. However, the importance of Aβ-associated oxidative stress for neurodegeneration relative to that of other forms of AD-associated oxidative stress still needs to be investigated.

Thus, in the current study, the combination of ferrous sulfate, Aβ1-42, and buthionine sulfoximine (BSO) (FAB) was chosen to recapitulate AD pathophysiology as closely as possible. Aβ1-42 is more amyloidogenic than other Aβ peptides due to its hydrophobic characteristics. Ferrous sulfate was used as a prooxidant factor to induce oxidative stress under conditions of glutathione synthase inhibition. Glutathione synthase is an important enzyme in the antioxidant system, and inhibition of this enzyme by BSO likely contributes significantly to AD pathogenesis. Several studies have shown that administration of the FAB cocktail into the ventricular system by a pump over 4 weeks can be used to successfully model AD in rats. In the current study, the FAB cocktail was administered to primary rat hippocampal cells. We cultivated mixed cells isolated from the rat hippocampus, which included both glial and neuronal cells. This allowed us to recapitulate the complex environment of the brain, including neuroglial interactions, which play a very important role in AD pathogenesis. This cellular model of AD allowed us to monitor dynamic metabolic changes over several days. This study mainly focused on the prooxidant aspects of AD pathogenesis. Furthermore, cellular bioenergetics was assessed and routine metabolic tests were performed to evaluated cell survival and viability.

## Materials and methods

### Study material and experimental design

All animal experiments were approved by the local institutional committee of Animal Welfare and performed in accordance with international law regarding the protection of animals and the Guide for the Care and Use of Laboratory Animals (National Institutes of Health publication 8th Edition, 2011) and in compliance with the ARRIVE guidelines for animals. Experiments were carried out on primary hippocampal cells obtained from adult rats. The cells were obtained from six hippocampal samples (from 3 animals) and divided into eight groups, with 5 wells for each group. The cells in groups 2–8 were treated with various agents that cause neuronal damage. Specifically, group 2 was treated with aggregated Aβ (Aβ1-42) at a final concentration of 5 μM, group 3 was treated with BSO at a final concentration of 4 mM, group 4 was treated with iron sulfate (Fe) at a final concentration of 0.33 mM, group 5 was treated with BSO + Fe, group 6 was treated with BSO + Aβ, group 7 was treated with Fe + Aβ, and group 8 was treated with BSO + Fe + Aβ. Group 1 served as the control group. All chemicals were obtained from Sigma (Sigma, USA) and were of the highest purity available.

### Cell culture

Primary hippocampal cells were isolated from the brains of two-month-old rats (100–150 g, female) as described previously^24,25^. Briefly, the animals were decapitated after being euthanized with CO_2_. The brains were isolated, and then the hippocampus was immediately collected. The hippocampus was mechanically dissociated using syringes with decreasing needle diameters. We obtained 0.2–0.4 × 10^6^ cells from each hippocampal sample. The hippocampal cells were cultured in NeuroCult neural cell culture medium (STEMCELL Technologies Inc., Vancouver, Canada) supplemented with 10% NeuroCult proliferation supplement, 10% fetal bovine serum and 1% penicillin/streptomycin. A total of 0.25 × 10^6^ cells/ml were plated in 0.5 ml of normal culture medium in each well of a 24-well plate precoated with poly-d-lysine (Sigma, USA). The hippocampal cells were then incubated in a humidified atmosphere at 37 °C in 5% CO_2_. Half of the medium was replaced with fresh media every 5 days. Cell viability was assessed using the trypan blue exclusion test. Experimental agents were added on the 5th day after cell seeding after the medium was refreshed (Day 0). This period served as an adaptation period for the cells. Tests were performed on the following days of culture: 0, 3, 7, 14, and 21.

### Cell microscopy and immunofluorescence assay

Live cell imaging and fluorescence microscopy were performed using an EVOS FL Cell Imaging System (Thermo Fisher Scientific, USA). Phase-contrast microscopy was applied to count unstained live cells and evaluate morphology. Immunofluorescence was performed on cells on #1.5 coverslips according to a previously described protocol^24^. Cells were plated on coverslips in culture dishes and cultured for 14 days (at 37 °C in 5% CO_2_ in a humidified incubator). The cells were fixed at the respective time points with 4% paraformaldehyde (PFA) for 15 min and then washed once with 200 µl of PBS (0.1 M, pH 7.4) for 5 min. The liquid was removed by a suction pump throughout the procedure. The cells in the six-well plates were washed three times for 5 min each with 1.5 ml of PBST (0.3% Triton X-100 in PBS). The fixed cells were preblocked with 1.5 ml blocking buffer (1% BSA, 4% normal goat serum, 0.3% Triton X-100 in PBS) for 1 h at RT. After blocking, 1.5 ml of primary antibody (glial fibrillary acidic protein (GFAP) or neuronal nuclei (NeuN), 1:1000) diluted in blocking buffer was added to each well. The plates were then incubated on a shaker at 100 rpm and 4 °C for at least 18 h. After incubation with primary antibody, the buffer was removed, and the cells were washed 3 × 10 min with 1.5 ml of PBST. Secondary antibody (Alexa Fluor 568-conjugated goat-anti-rabbit and Alexa Fluor 488-conjugated goat-anti-mouse, diluted 1:1000 in blocking buffer) was added to the wells. The cells were incubated for at least 1.5 h at RT. The plates were covered with aluminum foil to protect them from light. After incubation with secondary antibody, the coverslips were washed for 10 min in 1.5 ml of PBST and then washed twice for 10 min with 1.5 ml of PBS. The coverslips were held with fine forceps and dipped into 50 ml Falcon tubes containing Milli-Q H_2_O to rinse off the PBS. The nuclei were stained using DAPI. Before mounting, the coverslips were gently dried by touching the edge with tissue paper. The cells attached to the coverslips were mounted on Superfrost slides with 10 µl of mounting media. The slides were stored in the dark at 4 °C and protected from light until imaging. After immunohistochemistry, GFAP^+^/(DAPI^+^-GFAP^+^) and GFAP^+^/NeuN^+^ cells were counted.

### Biochemical tests

The levels of molecules reflecting cellular metabolism (glucose and lactate levels) and cell damage (lactate dehydrogenase (LDH) levels) in the medium were measured using Roche Cobas C311 and Cobas E411 automatic biochemical analyzers (Roche Cobas, Switzerland) and appropriate test kits. Protein carbonylation, advanced oxidation protein products (AOPP), and TBARS levels in the culture medium were measured to assess oxidative stress levels in the cultured cells. All spectrophotometric measurements were performed using a Thermo Scientific™ Multiskan™ GO Microplate Spectrophotometer (Thermo Fisher Scientific, USA). Biochemical tests were performed on 3, 7, 14, and 21 days of culture.

Protein carbonylation was assessed according to the method described by Levine et al.^26^ with minor modifications. Briefly, 10 µl of medium was mixed with 0.5 ml of 2,4-dinitrophenylhydrazine (20 mM), and the samples were vortexed for 1 h. Then, 1.0 ml trichloroacetic acid (20%) was added, and the samples were incubated for 15 min at room temperature. The mixtures were centrifuged at 3400 × g, and the precipitates were washed three times with an ethyl acetate:ethanol mixture (1:1). The protein pellets were dissolved in 3 ml of urea (8.0 M), and the absorbance was measured at 360 nm. The protein concentration was determined by measuring the absorbance at 280 nm. The carbonyl content was calculated using the molar extinction coefficient (22,000/M cm) and is expressed in terms of nmol mg^-1^ of protein.

Spectrophotometric determination of AOPP levels was performed according to Witko’s method^27^. Briefly, 50 µl of medium was mixed with 0.5 ml of potassium phosphate buffer (0.1 M, pH 7.4). Then, 50 µl of potassium iodide (1.16 M) was added to each tube. Fifty microliters of glacial acetic acid were added 2 min later. The absorbance of the reaction mixture was immediately read at 340 nm and compared with that of the blank (without sample). An additional blank (without potassium iodide) was used to subtract the absorbance of the sample itself. The results are expressed in terms of nmol AOPP mg^-1^ of protein.

Lipid peroxidation was evaluated by measuring the level of TBARS as described by Uchiyama and Mihara^28^. Briefly, 1.0 ml of orthophosphoric acid (2%) and 0.5 ml of thiobarbituric acid (0.8%) were added to 50 µl of culture medium. The mixture was heated in a boiling water bath for 45 min. After cooling, the resultant chromogen was extracted with 2.0 ml of n-butyl alcohol, and the organic phase was separated by centrifugation at 3000 × g for 10 min. The absorbance of the supernatant was measured at 535 and 580 nm. After subtracting the nonspecific absorbance (580 nm), the TBARS concentration was determined using the molar extinction coefficient (155/mM cm). The results were expressed in terms of nmol TBARS/mg of protein.

### Measurement of bioenergetics parameters

We seeded 0.25 × 10^6^ cells/ml in 80 µl of culture medium in each well of an XF HS Mini cell culture microplate (Seahorse Bioscience, Billerica, MA, USA) and incubated them overnight at 37 °C in 5% CO_2_. The culture medium was replaced with 180 µl of bicarbonate-free DMEM supplemented with 10 mM glucose, 1 mM pyruvate and 2 mM l-glutamine (pH 7.4). The cells were incubated at 37 °C for 30 min before measurement. The oxygen consumption rate (OCR) and extracellular acidification rate (ECAR) were measured using an XF HS Mini Extracellular Flux Analyzer. The following inhibitors were used at the indicated concentrations: 1.5 µM oligomycin, 2 µM FCCP, and 0.5 µM rotenone and antimycin A. Analyses were conducted using Wave software and XF Report Generators (Agilent Technologies). The sensor cartridge for the XFe analyzer was hydrated at 37 °C 1 day before the experiment. The OCR and ECAR were normalized to the total amount of protein. Three wells from each group were analyzed, and the average was obtained.

### Data analysis

Data analysis was carried out with GraphPad InStat software Version 3.10 (GraphPad Software, San Diego, CA). Unpaired *t test* was used to compare the levels of biomarkers between groups (n = 5). The normality of the data was assessed by the Kolmogorov–Smirnov test. The means, standard deviations (SDs) and associated P values are reported. Ps < 0.05 indicated statistical significance.

### Institutional review board statement

The experimental protocol was performed in accordance with the guidelines of the European Communities Council Directive (86/609/EEC) and was approved by the Ethics Committee of Yerevan State Medical University after Mkhitar Heratsi (Identification code N 4–2/18. Date: 15 November 2018).

## Results

### Cell number and morphology

**Figure 1:**
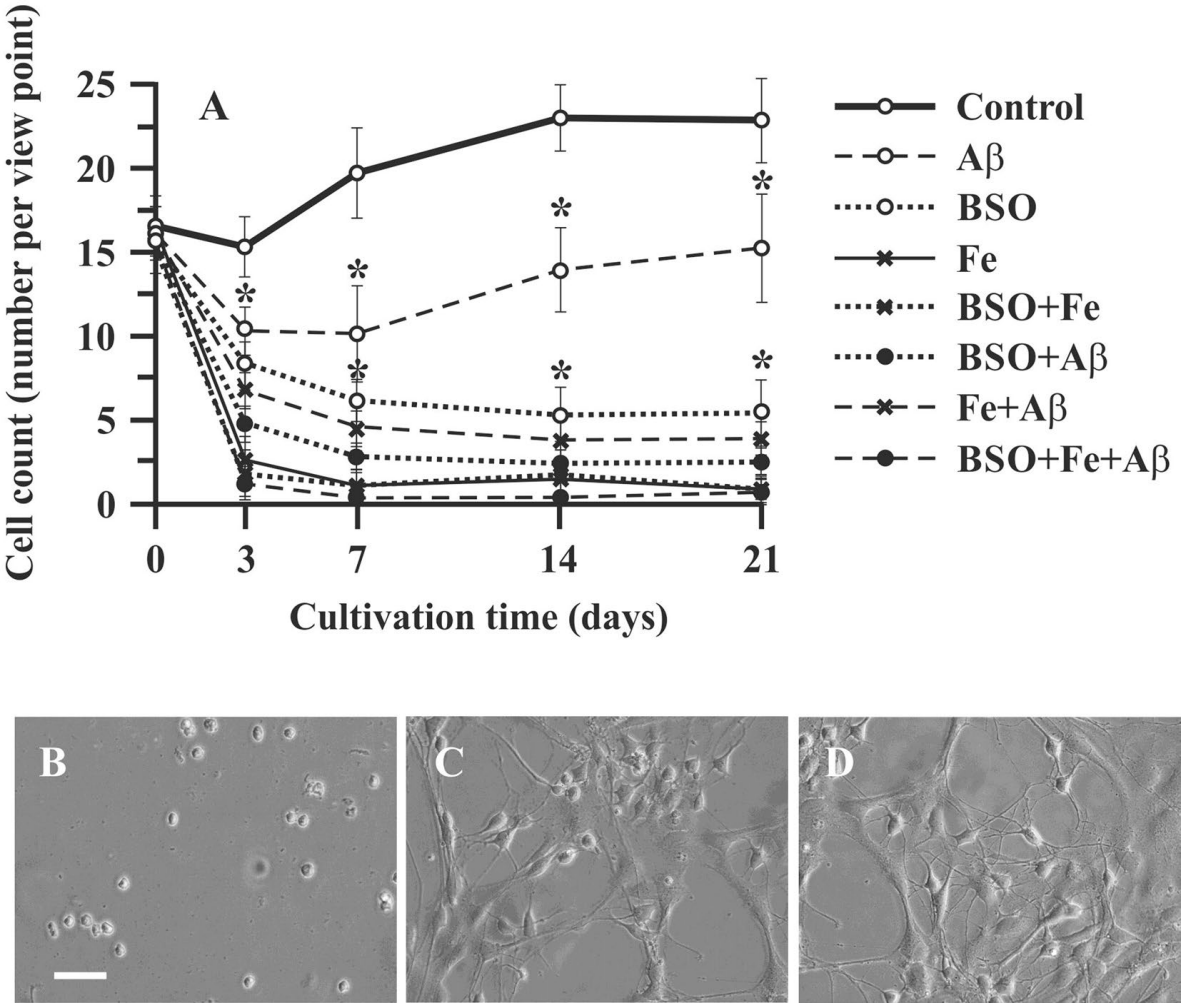
Effect of Aβ, BSO, and Fe2 + on neuronal cell count and morphology. (**A**) Changes in the number of primary rat hippocampal cells. (**B**) Neuronal cell morphology on the 7th day of culture. (**C**) Neuronal cell morphology on the 14th day of culture. (**D**) Neuronal cell morphology on the 21st day of culture. Scale bar, 100 μm. n = 10. * P < 0.05. The error bars indicate the mean ± SD. Hippocampal cells were isolated from the brains of two-month-old rats. The cells were cultured in NeuroCult medium supplemented with 10% NeuroCult proliferation supplement, 10% fetal bovine serum and 1% penicillin/streptomycin.

Hippocampal cells growth in primary culture (control cells) was characterized by a short lag phase during the first 7 days followed by a moderate increase in cell number (by 27.2%) until the 14th day of cultivation (exponential phase). The last 7 days of culture (14–21 days) was a stationary phase, during which we did not observe cell growth (Fig. 1A). The changes in neuronal morphology were evaluated using phase-contrast microscopy. Until the 7th day of culture, hippocampal cells appeared as round cells (Fig. 1B). After 7 days of culture, the hippocampal neurons extended moderate-sized apical dendrites, and early branches began to grow (Fig. 1C). On the 21st day of cultivation, although no proliferation was observed, extended axons and dendrites were observed (Fig. 1D).

Aggregated Aβ (1-42) had a marked effect on neuronal cell growth in the exponential phase (7–14 days) of growth in culture; on the 7th day of culture, the cell count in the Aβ group was 51% lower (P < 0.05) than that in the control group. After this point, cells in the Aβ group retained moderate proliferative capability, and the increase in cell number in this group in the exponential phase (from the 7th to 14th days of culture) was 21% (Fig. 1A). This growth rate was not significantly different from that in the control group (P > 0.05). The other experimental groups exhibited more pronounced cell loss and no proliferation. The cell count in these groups in the lag phase was markedly decreased, and no exponential growth phase was observed. The combination of BSO or Fe and Aβ decreased the cell count by 82% and 84%, respectively (P < 0.05). The combination of all compounds (Aβ, BSO, Fe^2+^) in one plate (the FAB model) had in much more detrimental effects. The cell number in the FAB group was only 6.5% of that in the control group (P < 0.05).

### Immunocytochemistry

**Figure 2:**
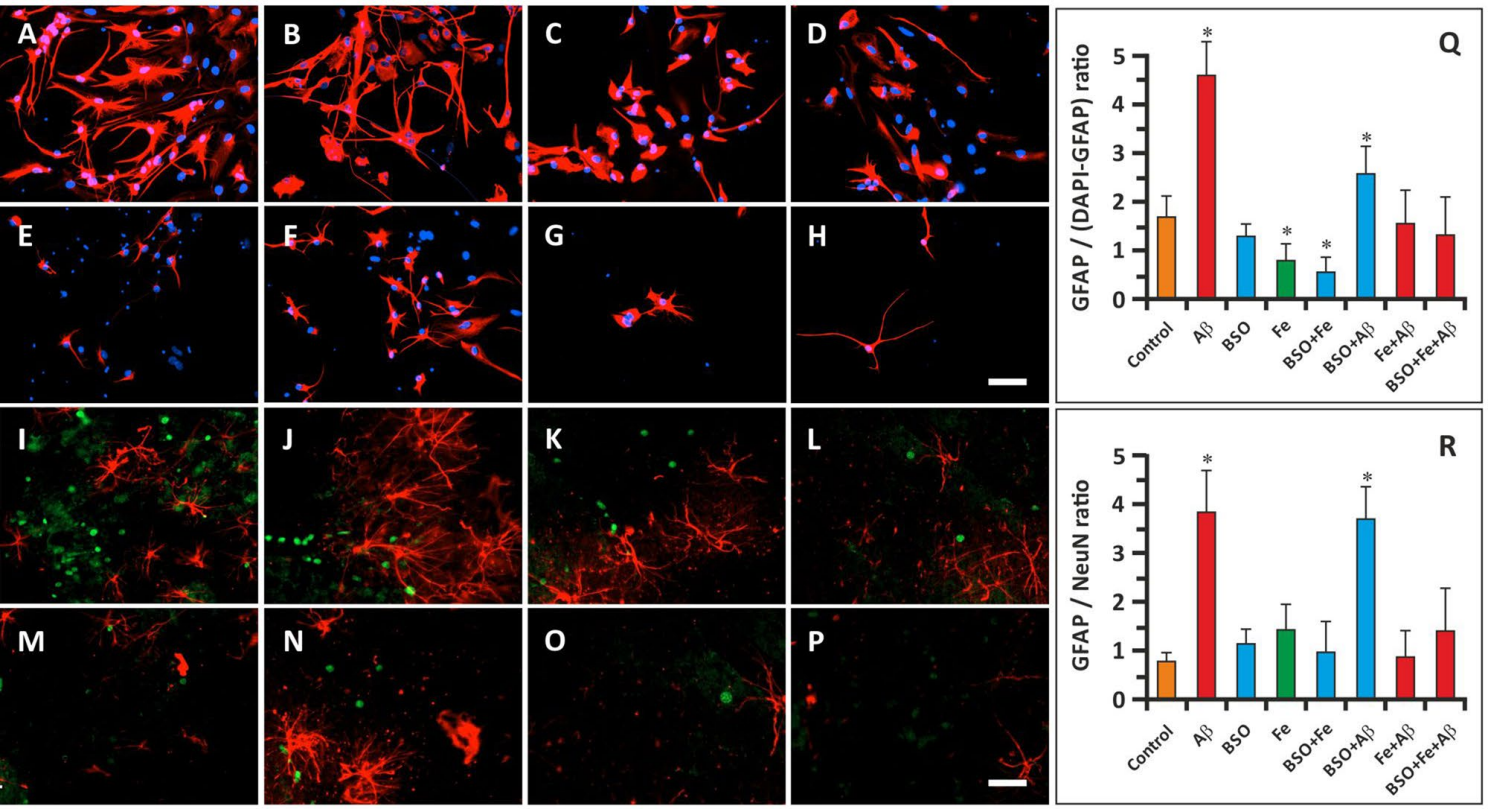
Effect of Aβ, BSO, and Fe^2+^ on the ratio of glial cells to neuronal cells and glial and neuronal morphology. (**A**–**H**) Immunostaining of GFAP (red) and DAPI staining (blue) in hippocampal cells on the 14th day of cultivation. Scale bar, 100 μm. (**I**–**P**) Immunostaining of GFAP (red) and NeuN staining (green) in hippocampal cells on the 14th day of cultivation. Scale bar, 200 μm. (**Q**) GFAP^+^/(DAPI^+^-GFAP^+^) cell ratio. (**R**) GFAP^+^/NeuN^+^ cell ratio. n = 10. * P < 0.05. The error bars indicate the mean ± SD.

In the current study, we did not purify neuronal cells but analyzed the effect of FAB on mixed hippocampal cells in culture. Therefore, in the first immunocytochemistry study, we mainly focused on hippocampal astroglia. To this end, we stained cells with an anti-GFAP antibody and DAPI. Immunocytochemistry was performed on the 14th day of cultivation in the exponential growth phase. We calculated the GFAP^+^/(DAPI^+^-GFAP^+^) ratio, which was 1.67 ± 0.34 in the control group (Fig. 2A, Q). This ratio was markedly increased in the cells treated with Aβ (Fig. 2B, F, Q); the GFAP^+^/(DAPI^+^-GFAP^+^) ratio was 4.57 ± 0.68 in the Aβ group and 2.50 ± 0.73 in the BSO + Aβ group (P < 0.05). In these groups, the cells retained the ability to form axons and dendrites. The number of GFAP^+^ cells in the Fe^2+^-treated groups was decreased, and the GFAP^+^/DAPI^+^-GFAP^+^ ratio was 0.78 ± 0.26 in the Fe group and 0.56 ± 0.22 in the BSO + Fe group (P < 0.05) (Fig. 2D, E, Q). Although the GFAP^+^/(DAPI^+^-GFAP^+^) ratio was normal in the BSO group (1.39 ± 0.41; P > 0.05), cells in this group exhibited diminished axon and dendrite formation (Fig. 2C, Q). GFAP^+^/DAPI^+^-GFAP^+^ ratio in the remaining groups was not significantly differ from the control samples (Fig. 2 G, H, Q).

In the next immunocytochemistry study, hippocampal cells were stained for GFAP and NeuN. The GFAP^+^/NeuN^+^ ratio in control samples was 0.69 ± 0.17 on the 14th day of cultivation (Fig. 2I, R). Similar to the data reported above, the GFAP/NeuN ratio was significantly increased in Aβ and BSO + Aβ samples (3.83 ± 0.87 and 3.75 ± 0.76, respectively) (P < 0.05) (Fig. 2J, N, R). The GFAP/NeuN ratio in the other groups did not differ significantly from that in the control group (Fig. 2K–M, O–R).

### Analysis of neuronal metabolism and survival via determination of glucose, lactate, and LDH levels

**Figure 3:**
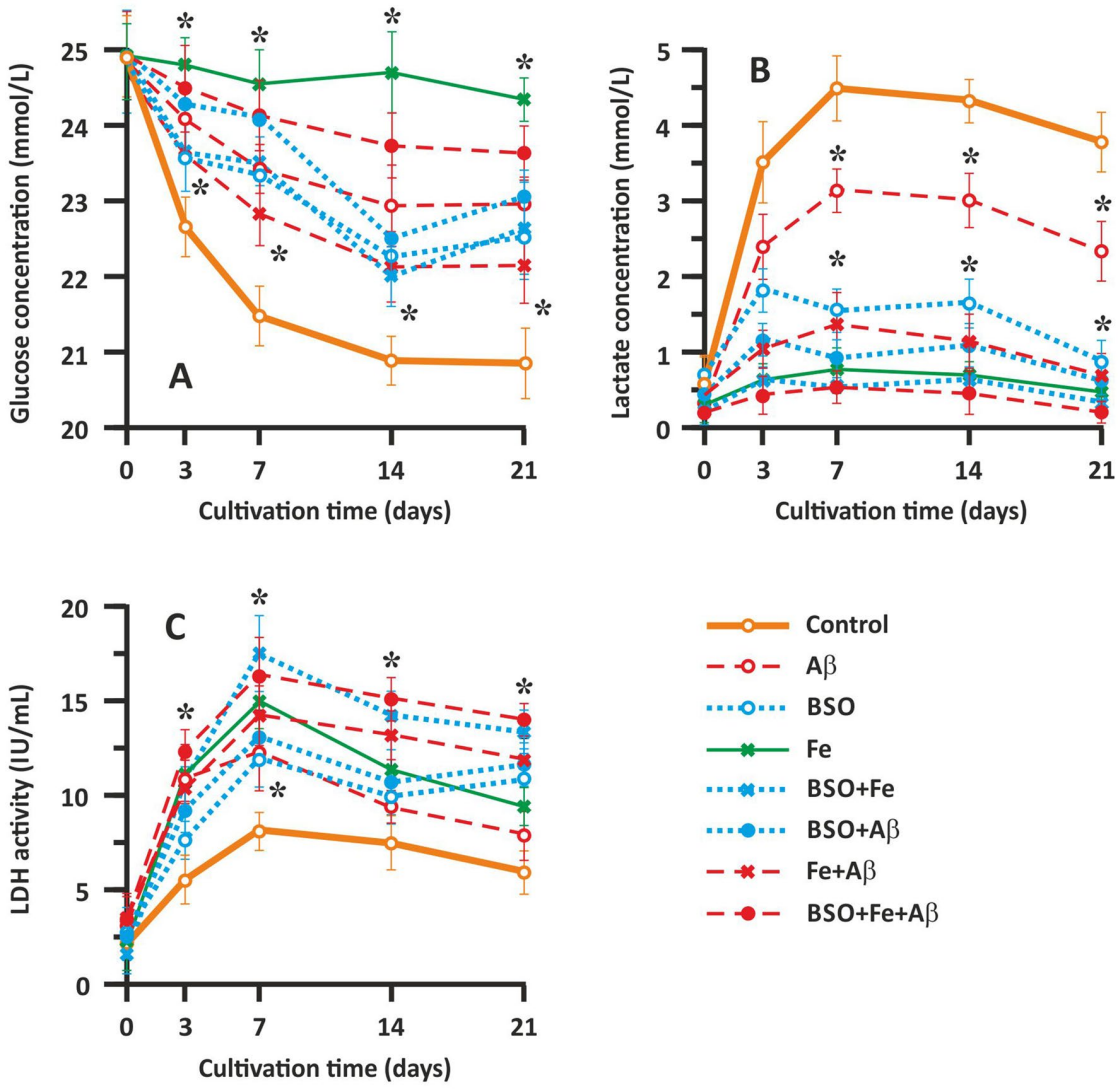
Effect of Aβ, BSO, and Fe^2+^ on hippocampal cell metabolism and survival. (**A**) Change in the glucose concentration in the culture medium. (**B**) Change in the lactate concentration in the culture medium. (**C**) Change in LDH activity in the culture medium. Biochemical parameters reflecting cellular metabolism (glucose and lactate levels) and cell damage (LDH activity) in the extracellular medium were measured using Roche Cobas C311 and Cobas E411 automatic biochemical analyzers (Roche Cobas, Switzerland) and appropriate test kits. n = 10. * P < 0.05. The error bars indicate the mean ± SD.

Glucose and lactate concentrations and LDH activity in the culture medium were measured to evaluate neuronal metabolism and damage, respectively (Fig. 3). We found that in the control samples, the glucose concentration gradually decreased until the 14th day of cultivation (Fig. 3A). The glucose content in the culture medium gradually decreased from 24.91 ± 0.52 to 21.03 ± 0.37 mmol/l in the control group. During the last week of cultivation (14–21 days of culture), the glucose concentration in the culture medium did not change significantly. The change in the glucose concentration was strongly correlated with the change in the cell count presented above and reflects cell metabolism, which gradually decreased. Fe application almost completely abolished glucose utilization, likely by causing oxidative stress and cell death (Fig. 3A). In the Aβ-treated groups (the Aβ, Fe + Aβ, and BSO + Fe + Aβ groups), intermediate toxicity was observed. Glucose utilization was most markedly affected in the BSO + Fe + Aβ group but less affected in Aβ group (Fig. 3A). The change in glucose content in the culture medium was similar between the Aβ group and the control group. Interestingly, wave-like fluctuations in glucose concentrations were observed in the BSO-treated groups (the BSO, BSO + Fe, and BSO + Aβ groups). Generally, the change in glucose concentrations in these groups was similar to that in the Aβ-treated groups, but there were two periods in which glucose utilization was abolished, i.e., 3–7 day of culture and 14–21 days of culture (Fig. 3A).

The change in lactate accumulation in the neuronal cell medium was opposite of the change in glucose concentrations, which is expected and normal for metabolically active cells. In the control group, we observed lactate accumulation until the 7th day of culture, when the analyte concentration reached 4.51 ± 0.49 mmol/l (Fig. 3B). The lactate concentration in the control group gradually decreased after the 7th day and until the end of cultivation and finally reached a value of 3.84 ± 0.42 mmol/l. The change in lactate accumulation in the Aβ-treated groups (the Aβ, Fe + Aβ, and BSO + Fe + Aβ groups) resembled the change in glucose concentrations. The curves representing the change in the lactate concentration in these groups showed the same shape as the curve of the control group, but the concentrations were lower. Cells treated with Aβ alone showed less marked changes, with lactate concentrations reaching the maximal value (3.12 ± 0.27 mmol/l) on the 7th day of cultivation and gradually decreasing to 2.44 ± 0.35 mmol/l (Fig. 3B). In the BSO-treated groups (the BSO, BSO + Fe, and BSO + Aβ groups), the lactate concentration reached the maximal value on the 3rd day of culture and then exhibited wave-like fluctuations until the 21st day of culture (Fig. 3B).

Intracellular LDH release indicates a change in cell membrane permeability and reflects the extent of damage. In our experiments, a gradual increase in LDH activity was observed in the control group until the 7th day of cultivation, with LDH activity peaking at 7.86 ± 0.48 IU/ml. Afterward, LDH activity gradually decreased, reaching a value of 6.03 ± 0.76 IU/ml at the end of cultivation (Fig. 3C). Overall, the change in LDH activity resembled the change in lactate levels and reflected cellular metabolism. The change in LDH activity in the Aβ-treated groups (the Aβ, Fe + Aβ, and BSO + Fe + Aβ groups) resembled the change in LDH activity in the control group, but LDH activity was much higher in the control group than in the other groups. The greatest degree of cellular damage was observed in the BSO + Fe + Aβ group, with LDH activity reaching 16.07 ± 1.93 IU/mL (P < 0.05) on the 7th day of cultivation (Fig. 3C). In the BSO-treated groups (the BSO, BSO + Fe, and BSO + Aβ groups), LDH activity was decreased on the 14th day of cultivation, which is consistent with the results of previous metabolic tests.

### Oxidative modification of cellular proteins and lipids

**Figure 4:**
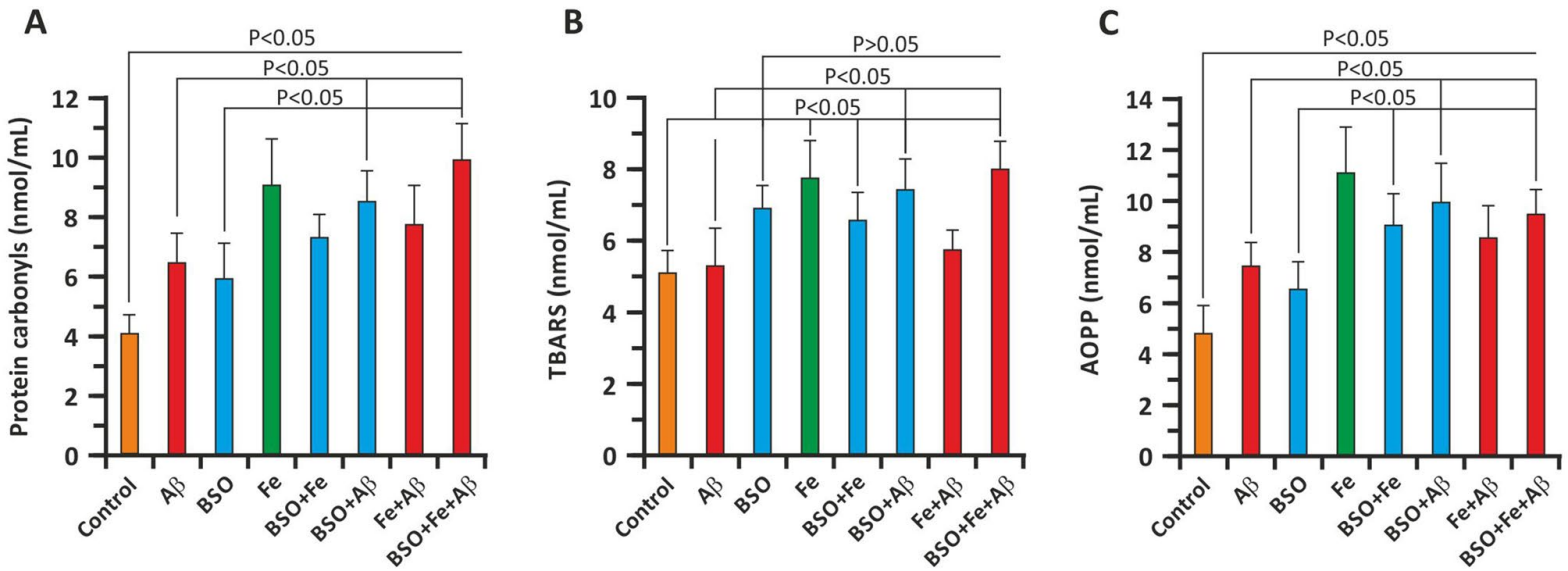
Effect of Aβ, BSO, and Fe^2+^ on indicators of oxidative damage in hippocampal cells. (**A**) Carbonylation of cellular proteins. (**B**) Lipid oxidation product (TBARS) levels. (**C**) AOPP levels. Oxidative parameters were measured in neuronal cell lysates on the 3rd day of culture. n = 10. The error bars indicate the mean ± SD.

To address the prooxidative capacity of Aβ and FAB, we measured several relevant parameters. On the 3rd day of culture, we measured protein carbonylation, lipid peroxidation (TBARS), and AOPP levels in the culture medium. The findings showed that Aβ significantly enhanced protein oxidative modification (Fig. 4A, C). In the Aβ group, the protein carbonylation level was 6.43 ± 0.94 nmol/ml and the AOPP concentration was 7.40 ± 0.89 nmol/ml, whereas in the control group, the protein carbonylation level and AOPP level were 4.02 ± 0.71 nmol/ml and 4.80 ± 1.07 nmol/ml, respectively (P < 0.05 versus the Aβ group). Further addition of BSO and BSO + Fe significantly increased in oxidative damage to cellular proteins. The protein carbonylation level was 8.49 ± 1.21 nmol/ml and 9.98 ± 1.54 nmol/ml in the BSO + Aβ and BSO + Fe + Aβ groups, respectively (P < 0.05). The AOPP level was also significantly increased. In the BSO + Aβ and BSO + Fe + Aβ groups, the AOPP level was 10.00 ± 1.38 nmol/ml and 9.98 ± 0.79 nmol/ml, respectively (P < 0.05 versus the Aβ group). Interestingly, Fe + Aβ resulted in less protein oxidation than Fe alone (Fig. 4A, C). BSO induced less protein oxidation in hippocampal neurons than Aβ. Additionally, Fe and Aβ enhanced the prooxidant effects of BSO. Similar to Aβ + BSO, Fe + BSO resulted in less oxidation than Fe alone (Fig. 4A, C).

As stated above, the degree of lipid peroxidation was evaluated by measuring TBARS content in neuronal cell lysates. The TBARS content in control cell lysates was 5.10 ± 0.67 nmol/ml (Fig. 4B). Compared to protein oxidation (carbonylation and AOPP formation), Aβ did not significantly alter TBARS levels (5.33 ± 1.12 nmol/ml; P > 0.05). The same was not true for BSO, which significantly enhanced lipid peroxidation in neuronal cell lysates (6.97 ± 0.89 nmol/ml; P > 0.05). Aβ + BSO and BSO + Fe, but not Fe alone, significantly increased TBARS levels (Fig. 4B). Here, we also observed an unexpected decrease in lipid peroxidation in the BSO + Fe and Aβ + Fe groups compared with the Fe group (Fig. 4B).

### Cellular bioenergetics analysis

**Figure 5:**
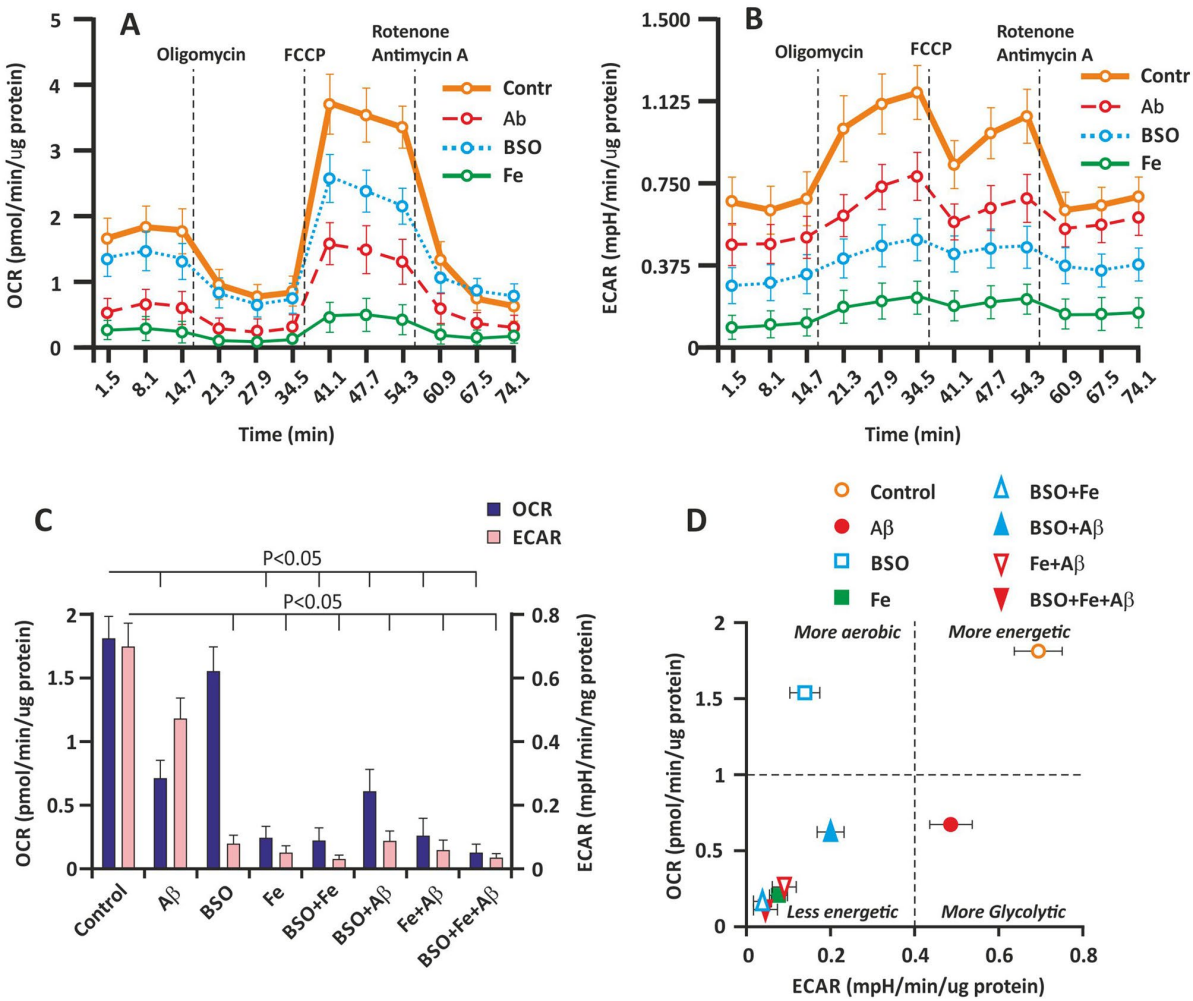
Effect of Aβ, BSO, and Fe^2+^ on bioenergetics parameters in hippocampal cells. (**A**) The OCR of cultured hippocampal cells on the 3rd day of culture. (**B**) The ECAR of cultured hippocampal cells on the 3rd day of culture. (**C**) Basal OCR and ECAR. (**D**) Metabolic profiles of hippocampal cells. Analyses were conducted using Wave software and XF Report Generators (Agilent Technologies).

To determine how Aβ, BSO, Fe, and their combination (FAB) affect cellular bioenergetics, we determined the OCR and ECAR by extracellular flux analysis. Cellular bioenergetics indicators were measured on the 3rd day of culture. The basal and ATP generation-coupled OCR and maximal respiration were significantly affected by Aβ and Fe (P < 0.05). However, the effect of BSO on OCR was negligible (Fig. 5A). BSO did not have a significant effect on the basal and ATP generation-coupled OCR (P > 0.05). Nevertheless, maximal respiration in the BSO group was almost 30% less (P < 0.05) than that in the control group. The Fe-treated groups exhibited the lowest basal OCR (Fig. 5C).

The ECAR showed the opposite change. Using extracellular flux analysis, we found that BSO and Fe markedly decreased the ECAR; however, the effect of Aβ on the ECAR was less obvious (Fig. 5B). The BSO- and Fe-treated groups exhibited the lowest basal ECAR (Fig. 5C).

The basal ECAR and OCR were plotted, and the results are shown in Fig. 2C. The data represent the change in the metabolic profile of the cells induced by Aβ, BSO, and Fe. The different effects of Aβ and BSO on cellular bioenergetics are shown. The Aβ-treated groups showed a more glycolytic profile, and a more aerobic profile was observed in the BSO-treated groups (Fig. 5D). Aβ and BSO exerted a synergistic effect, resulting in a less energetic cellular phenotype due to a decrease in both aerobic and glycolytic flux in the cell. Fe overload more markedly inhibited cellular bioenergetics (Fig. 5D).

## Discussion

In the current study, we show for the first time the effect of Aβ and the components of FAB on growth parameters, oxidative damage, and bioenergetics parameters in adult rat mixed primary hippocampal cells. First, we characterized the changes in cell number and morphology in the control group. The control group exhibited a classical growth curve: a lag phase during the first 7 days, an exponential phase (7–14 days), and a stationary phase (14–21 days of culture). Most previous studies have focused on keeping adult neurons alive for a period of time (21 days)^25,29,30^.

In the lag phase, hippocampal cells were round cells without outgrowths. In the exponential phase, hippocampal neurons exhibited moderate-sized apical dendrites, and early branches began to grow. In the stationary phase, despite loss of cell proliferation, extended axons and dendrites were observed. These data are in good accordance with previous reports^25^.

Aggregated Aβ (Aβ1-42) had a marked effect on the changes in neuronal cell growth in the exponential phase. Aβ decreased the number of cells (by 51%) on the 7th day of culture. After this, cells in the Aβ group retained moderate proliferative capability. The other experimental groups exhibited more pronounced cell loss and no proliferation. The cell number in the lag phase was robustly decreased in these groups, and no exponential phase was observed. Aβ + BSO and Aβ + Fe decreased the cell number by 82% and 84%, respectively. The BSO + Fe + Aβ group (FAB model) showed maximum inhibitory/cytotoxic activity and resulted in much more detrimental/cytotoxic consequences.

The effect of Aβ on cultured hippocampal neurons was shown previously and can be explained by Aβ-mediated neuronal cell death resulting from the loss of full-length Tau and/or the generation of toxic fragments^31^. The effect of chronic intracerebroventricular delivery of Aβ in combination with the prooxidant Fe^2+^ or the glutathione synthesis inhibitor BSO on brain histopathology and memory loss in adult rats was shown previously^32^. The destructive effect of Aβ in combination with oxidative stress induced by BSO and Fe most appropriately reflects AD-like symptomatology. However, there have been no studies on the effect of FAB in cultured neurons.

Using immunocytochemistry (with anti-GFAP and anti-NeuN antibodies), we assessed neuronal cell loss in the Aβ-treated groups and membrane damage/loss in the BSO- and Fe-treated groups. FAB caused total cell death. Our results were in good accordance with previous in vivo experiments^33–36^.

We also showed the detrimental effect of Aβ and other FAB components on metabolism. It was found that glucose consumption and lactate production were inhibited. Interestingly, unique changes in the levels of glucose and lactate were observed in the BSO-treated groups. The maximal decrease in the levels of these metabolites was observed on the 7th day of culture, after which the changes were relatively similar to those in the control group. The detrimental effect of BSO can be explained by its ability to deplete glutathione, which causes apoptotic cell death^37^. This phenomenon was not observed in the Aβ-treated groups and might have been due to the purification of BSO from the medium after replacement of irreversibly inhibited enzymes with new enzymes. This was not true for the Aβ-treated samples, probably because Aβ binds to cellular membranes, as presented earlier,this binding allows a constant Aβ concentration to maintained at all cultivation time points despite medium replacement^38^.

We analyzed the prooxidant activity of the studied analytes and found an increase in lipid peroxidation in the BSO-treated groups (high TBARS level) and oxidative damage to proteins in the Aβ-treated groups (high levels of protein carbonylation and AOPP). These findings support in the immunocytochemistry results, indicating that the membrane was likely damaged or lost in the BSO-treated groups, and are consistent with the literature^39^.

Finally, the difference in the effects of Aβ and BSO were assessed by extracellular flux analysis. We observed that Aβ inhibited of mitochondrial oxidative phosphorylation and that BSO inhibited glycolysis. The combination of these resulted in maximal alterations in cellular bioenergetics.

Our findings clearly show that aggregated Aβ1-42 alone is significantly less toxic to hippocampal cells. Aggregated Aβ damages neurons, and glial cells proliferate to remove Aβ from the hippocampus. External prooxidant agents (Fe^2+^) or inhibition of the internal antioxidant defense by BSO is more toxic to hippocampal cells than aggregated Aβ alone. Hippocampal cells fight against Aβ-induced damage more effectively than against oxidative damage. However, the combination of Aβ, external oxidative damage and inhibition of internal antioxidant defense is even more toxic, impairs cellular defense systems, and may represent the late phase of AD-associated cell damage.

Immunohistochemical analysis clearly indicated that Aβ targeted neuronal cells (NeuN positive). Glial cells proliferate and can remove toxic Aβ from the cellular environment; This explains why we observed and increase in the number of GFAP^+^ relative to NeuN^+^ cells. Aβ exerted prooxidant activity, as evidenced by increased protein carbonylation and AOPP levels. It is likely that Aβ-dependent oxidative damage might have been due to inhibition of mitochondrial oxidative phosphorylation by Aβ, which was observed in our experiments using extracellular flux analysis.

It seems that Aβ toxicity mechanism is based on ROS accumulation. All around ROS and metals with transparent valency. First of all, the whole Aβ molecule has some metal-binding sites in its first 15 amino acids constituted by the histidines 6, 13, and 14 and the tyrosine in the 10 position. These all have well-known and powerful metal-binding sites, and a nearby affinity to the best metallic chelants currently known^40^. The Aβ possesses the ability to reduce Cu^2+^ and Fe^3+^ towards Cu^+^ and Fe^2+^, respectively. This way, the molecular oxygen can react with reduced metals thus generating superoxide anion, which in turn combines with two hydrogen atoms to form hydrogen peroxide that may later react with another reduced metallic ion and then forming the hydroxyl radical by Fenton reaction. The Aβ (in its radical form) can extract protons from the neighboring lipids or proteins, thus generating lipid peroxides and carbonyls, respectively^41^. Above it, using data achieved by extracellular flux analysis, we clearly demonstrated Aβ induced mitochondrial dysfunction. The later can be considered as a next cause of ROS overproduction in neurons. Growing evidence suggests that Aβ has deleterious effects on mitochondrial function and contributes to energy failure, neuronal apoptosis and production of ROS in AD brain^42^. However, in all probability, well-functioning antioxidant defense system of the organism is able to compensate prooxidant consequences of Aβ alone. Thus, as a real and powerful cause of Alzheimer’s disease-like neurodegeneration, we consider simultaneous damage of pro/antioxidant homeostasis with the accumulation of Aβ peptides in neuronal cell.

## Conclusion

In conclusion, the results of this study show that the combination of aggregated Aβ, an antioxidant-depleting agent (BSO) and a prooxidant (Fe^2+^) (FAB) more completely recapitulates neuronal cell damage in vitro. This strongly indicates a critical role for the combination effect of Aβ and oxidative stress in the development of neurodegeneration.

## Data Availability

Data can be made available by the corresponding author upon reasonable request.
