# Author Correction: The synergy of β amyloid 1-42 and oxidative stress in the development of Alzheimer’s disease-like neurodegeneration of hippocampal cells

**DOI:** 10.1038/s41598-022-25888-7

**Published:** 2022-12-14

**Authors:** Gohar Karapetyan, Katarine Fereshetyan, Hayk Harutyunyan, Konstantin Yenkoyan

**Affiliations:** 1 Neuroscience Laboratory, Cobrain Center, Yerevan State Medical University named after M. Heratsi, 2 Koryun Str., 0025 Yerevan, Armenia; 2 Department of Biochemistry, Yerevan State Medical University named after M. Heratsi, Yerevan, Armenia

Correction to: *Scientific Reports* 10.1038/s41598-022-22761-5, published online 25 October 2022

The original version of this Article contained an error in the Funding section.

“This work was supported by the State Committee of Science RA (21T-3A327, 20TTCG-3A012 and N 10-14/I-1) and the EU-funded H2020 COBRAIN project (857600).”

now reads:

“This work was supported by the State Committee of Science RA (21T-3A327 and N 10-14/I-1) and the EU-funded H2020 COBRAIN project (857600).”

The original Article has been corrected.

